# Coupled Motions Direct Electrons along Human Microsomal P450 Chains

**DOI:** 10.1371/journal.pbio.1001222

**Published:** 2011-12-20

**Authors:** Christopher R. Pudney, Basile Khara, Linus O. Johannissen, Nigel S. Scrutton

**Affiliations:** 1 Manchester Interdisciplinary Biocentre, Faculty of Life Sciences, University of Manchester, Manchester, United Kingdom; 2 Manchester Interdisciplinary Biocentre, School of Chemical Engineering and Analytical Sciences, University of Manchester, Manchester, United Kingdom; Case Western Reserve University School of Medicine, United States of America

## Abstract

Protein domain motion is often implicated in biological electron transfer, but the general significance of motion is not clear. Motion has been implicated in the transfer of electrons from human cytochrome P450 reductase (CPR) to all microsomal cytochrome P450s (CYPs). Our hypothesis is that tight coupling of motion with enzyme chemistry can signal “ready and waiting” states for electron transfer from CPR to downstream CYPs and support vectorial electron transfer across complex redox chains. We developed a novel approach to study the time-dependence of dynamical change during catalysis that reports on the changing conformational states of CPR. FRET was linked to stopped-flow studies of electron transfer in CPR that contains donor-acceptor fluorophores on the enzyme surface. Open and closed states of CPR were correlated with key steps in the catalytic cycle which demonstrated how redox chemistry and NADPH binding drive successive opening and closing of the enzyme. Specifically, we provide evidence that reduction of the flavin moieties in CPR induces CPR opening, whereas ligand binding induces CPR closing. A dynamic reaction cycle was created in which CPR optimizes internal electron transfer between flavin cofactors by adopting closed states and signals “ready and waiting” conformations to partner CYP enzymes by adopting more open states. This complex, temporal control of enzyme motion is used to catalyze directional electron transfer from NADPH→FAD→FMN→heme, thereby facilitating all microsomal P450-catalysed reactions. Motions critical to the broader biological functions of CPR are tightly coupled to enzyme chemistry in the human NADPH-CPR-CYP redox chain. That redox chemistry alone is sufficient to drive functionally necessary, large-scale conformational change is remarkable. Rather than relying on stochastic conformational sampling, our study highlights a need for tight coupling of motion to enzyme chemistry to give vectorial electron transfer along complex redox chains.

## Abstract 2

Directional electron transfer through biological redox chains can be achieved by coupling reaction chemistry to conformational changes in individual redox enzymes.

## Author Summary

Enzymes are proteins that catalyze a large array of chemical reactions, often in partnership with other enzymes. We understand in detail the chemical mechanisms of many of these reactions; however, the importance of the physical movements of enzymes during catalysis (or protein dynamics) is, increasingly, becoming apparent. In this study, we have placed fluorescent markers on an enzyme called cytochrome P450 reductase (CPR) to probe the dynamic changes in the physical conformation of the protein as the reaction chemistry proceeds. CPR catalyses the transfer of electrons from a small molecule donor (called NADPH), ultimately passing them to their partner enzymes called CYPs. We were able to correlate specific conformational changes with distinct chemical steps in CPR. We found that the chemical transformation itself induces the enzyme to adopt conformations that are required for its efficient interaction with CYPs. These findings have allowed us to develop a model of CPR activity in which electron transfer along the pathway from NADPH through CPR to CYP is tightly integrated with physical conformational control of the enzyme.

## Introduction

The relationship between dynamics and the function of proteins is important. Proteins undergo a wide range of motions in terms of time (10^−12^ to >1 s) and distance (10^−2^ to >10 Å) scales and any of these may be significant catalytically and related directly to function [1]–[5]. Proteins exist in an equilibrium of conformational states that define a multi-dimensional free energy landscape, enabling proteins to explore high energy states [6]. Mutagenesis can induce altered landscapes leading to energy traps with consequent effects on catalytic efficiency [7],[8]. It is in the nature of catalysis that high energy states are populated transiently during the course of an enzyme-catalyzed reaction. The ability to study these states experimentally, and to assess their impact on biological function, is a major challenge. Evidence points to a range of spatial and temporal dynamical contributions to substrate binding, product release, and chemical catalysis [9]–[11].

There is evidence supporting a role for domain motion in catalysis in the important family of diflavin oxidoreductases typified by human cytochrome P450 reductase (CPR) and human methionine synthase reductase (MSR) [12],[13]. Pulsed Electron Electron Double Resonance (PELDOR) studies of both CPR and MSR indicate landscape remodeling induced by ligand binding. Domain motion in this enzyme family has also been inferred from structural studies (crystallographic [14]–[17] and solution state [18]) and from pressure-dependent kinetic studies of electron transfer in CPR [12]. CPR is a membrane-bound NADPH-dependent oxidoreductase that contains FAD and FMN cofactors housed in discrete redox domains separated by a flexible hinge region [15]. CPR catalyzes electron transfer from NADPH to cytochrome P450 (CYP) enzymes in the endoplasmic reticulum. The relative orientation of the two flavin redox domains is variable, giving rise to “open” and “closed” conformations of the enzyme as seen in crystallographic analysis of homologous wild-type and mutant forms [16],[19]. NMR and small angle X-ray scattering studies suggest that CPR adopts a more closed conformation on coenzyme binding [18], similar to the conformation of crystallized rat CPR in which the dimethylbenzene moieties of the FAD and FMN cofactors are juxtaposed [15]. This closed conformation is optimal for interflavin electron transfer since the short interflavin distance enhances electronic coupling. Despite this close approach, interflavin electron transfer is slow (∼50 s^−1^) as measured by temperature jump [20],[21] and flash photolysis [22] time-resolved spectroscopies. These studies imply adiabatic control of electron transfer through conformational sampling [23]. This is consistent with temperature [24], pressure [12], and viscosity dependence [20] analysis of electron transfer kinetics, and with the multiple conformational states of human CPR seen in PELDOR studies [12]. Whilst the closed state of CPR is optimal for interflavin electron transfer, interaction with CYP enzymes requires a more open state. FMN domain residues that interact with CYP enzymes are occluded in the closed state [25]. A sequential opening and closing of CPR during the catalytic cycle is therefore proposed to facilitate internal electron transfer and subsequent transfer of electrons to CYP enzymes [16],[26]. This proposed cycling between open and closed conformations is consistent with impaired CYP reduction by CPR containing a non-native disulphide bond that links the FAD and FMN domains and the rescue of activity following reduction of this bond [27].

Evidence for conformational cycling during CPR catalysis is largely circumstantial. A direct means of analyzing conformational variations during enzyme catalysis is required to link the kinetics (and energy barriers) of conformational change to the chemical (redox) changes that result from hydride transfer (NADPH→FAD) and electron transfer (FAD→FMN). There are major problems to be addressed, including (i) identification of the “drivers” that open and close CPR; (ii) discrimination between electron transfer mechanisms that rely on conformational change coupled to chemical or binding events, or stochastic sampling of conformational space (i.e., conformational sampling mechanisms of electron transfer [28],[29]); (iii) whether the timescales for opening and closure support directional electron transfer from NADPH to CYP enzymes. With these key questions in mind our strategy has been to develop a direct method for analyzing the spatial and temporal properties of domain motion in human CPR using time-resolved Fluorescence Resonance Energy Transfer (FRET) during catalytic turnover. Our approach employs extrinsic fluorophores (Alexa 488 and Cy 5) attached at different positions on the solvent exposed surface of CPR to enable spatial (range ∼20–80 Å) and temporal (range ms to s) mapping of conformational variation during stopped-flow studies of flavin reduction by NADPH. In this way, we have been able to correlate the time dependence and extent of conformational change with individual rate constants for hydride and electron transfer in CPR. Using this direct approach, we have elucidated how motions link to enzyme chemistry, identified the “drivers” of these motions, and gained important new insight into how these motions facilitate directional transfer of electrons along human microsomal P450 chains.

## Results and Discussion

### An Experimental System That Reports Directly on CPR Domain Motion

**Figure 1:**
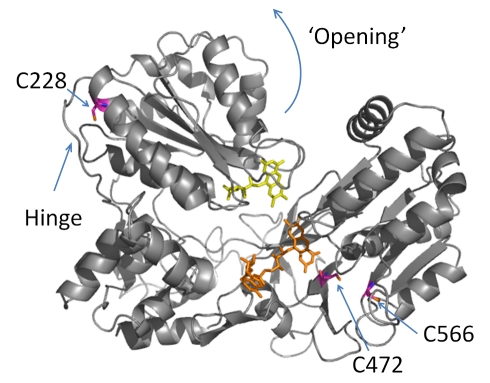
Homology model of CPR in the closed conformation. FMN is shown in yellow and FAD in orange. The curved arrow indicates the putative direction of domain motion as flavin reduction proceeds. The positions labeled with the extrinsic fluorophores are shown in magenta.

We generated homology models for several closed and open structures of human CPR based on X-ray crystal structures of the homologous rat CPR (94% sequence identity) [15],[16]. The fully closed structure is shown in Figure 1. Based on these models we reasoned that CPR can bind at least two mole equivalents of an extrinsic fluorophore through a thiol linkage to cysteine residues. Figure S1 shows the absorbance spectra for the donor (Alexa 488 (D)) and acceptor (Cy 5 (A)) fluorophores attached to cysteine residues, in fluorophore-labeled CPR (CPR-DA). The fluorophore and protein concentrations determined from this spectrum indicate stoichiometric attachment of the two fluorophores, giving a total fluorophore∶CPR ratio of 2∶1. Mass spectral analysis indicates that three cysteines are labeled using our protocol, namely C228, C472, and C566 (Figure S2), suggesting fractional labeling of each cysteine (see Text S1 for detailed discussion). C228 is located in the FMN domain and C472/C566 in the FAD domain, as shown in Figure 1. We have not attempted to remove the multiple cysteines in the FAD domain as we wish to study the wild-type enzyme, particularly since mutagenesis may have unknown effects on the protein dynamics. We note that there is only one labeled residue in the FMN domain, C288. From our homology models, opening of CPR results in a decreased distance between C228 in the FMN domain and C472/C566 in the FAD domain.

**Figure 2:**
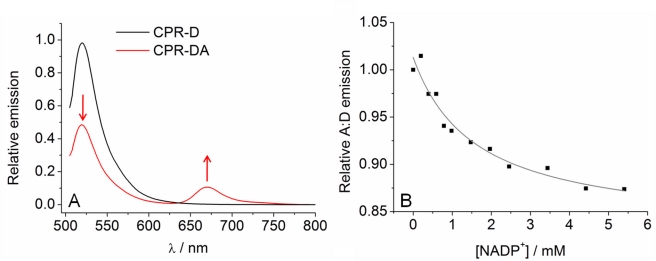
Coenzyme binding causes CPR to close. (A) Fluorescence emission spectra of CPR-D (black line) excited at 495 nm and CPR-DA excited at 495 nm (red line). The introduction of an acceptor fluorophore causes a decrease in donor emission and an increase in acceptor emission when the donor is excited (red arrows), which is demonstrative of FRET. The same concentration of donor fluorophore is present in both spectra. (B) Effect of titrating NADP^+^ on the relative change in FRET efficiency (expressed as variation in A∶D ratio). The solid line shows the fit to Equation 1. Conditions: 50 mM potassium phosphate pH 7, 25°C, 0.3 µM CPR-D, 0.6 µM CPR-DA.

Figure 2A shows the emission spectra of both donor labeled CPR (CPR-D) and CPR-DA, where the donor is excited at 495 nm. For CPR-DA, there is significant emission arising from the acceptor (∼670 nm) with a corresponding decrease in the emission arising from the donor (∼520 nm) compared to CPR-D. This indicates that there is efficient FRET from donor to acceptor when bound to CPR. We observed a small emission peak at ∼670 nm for the Cy 5 labeled CPR (CPR-A) when excited at 495 nm (Figure S3), but the relative emission is far smaller (<∼3%) than that attributed to FRET. If the FMN domain moves significantly relative to the rest of CPR (as is proposed to occur following flavin reduction), the FRET efficiency is expected to change, manifesting as a change in the ratio of acceptor to donor emission (A∶D). We were able to determine contributions to the FRET signal from inter-protein FRET as well as direct physical interaction of the extrinsic fluorophores with the flavin cofactors. A description of these control studies is given in Supporting Information (Text S1; Figure S4). We found no evidence to indicate that either of these processes contributes to the observed emission that we attribute to intraprotein FRET. We are therefore confident that the experimental setup reports on conformational change.

Several studies have suggested that CPR undergoes a conformational change associated with coenzyme binding [12],[18],[20],[30]. Specifically, PELDOR spectroscopy of di-semiquinoid CPR (containing FAD semiquinone and FMN semiquinone) has revealed that binding of NADP^+^ leads to formation of a more closed distribution of CPR structures compared to ligand-free di-semiquinoid enzyme [12]. It is possible to form an enzyme-coenzyme complex by incubating oxidized CPR with NADP^+^. Should binding of NADP^+^ induce CPR closure, the distance between C228 and the cysteines in the FAD domain will increase (as discussed above), resulting in poorer FRET efficiency between donor and acceptor (i.e., a decrease in the A∶D emission ratio). Figure 2B shows the resulting A∶D ratio for the emission of the CPR-DA fluorophores excited at 495 nm when titrated against NADP^+^. The individual donor and acceptor emission titrations are shown in Figure S5, normalized for the corresponding changes in fluorescence of CPR-D and CPR-A as described in Materials and Methods. This removes effects such as quenching by aromatic residues/NADP^+^ and FRET involving the flavin cofactors, leaving only changes attributable to FRET between the extrinsic fluorophores. From Figure 2B, the A∶D ratio decreases with increasing NADP^+^ concentration and saturates with a constant, *K*_S_ = 1.6±0.5 mM. These data indicate that coenzyme binding induces formation of a more closed form of CPR and demonstrate that our experimental system can detect relative domain movements in CPR.

### Conformational Change Occurs on the Timescale of Chemical Turnover

By monitoring the change in fluorescence emission of the fluorophores in stopped-flow studies of flavin reduction by NADPH, we have been able to correlate the kinetics of conformational change with enzyme chemistry. We assessed the degree of photo-bleaching of the fluorophores in oxidized CPR-D (ex 495 nm) and CPR-A (ex 655 nm). Example traces are given in Figure S6A. In each case there is a small decrease in fluorescence emission of ∼1% over 500 s. This small amount of photo-bleaching is not used to correct subsequent traces as the magnitude of the quenching is relatively small. Next, we determined if binding of NADP^+^ in stopped-flow studies causes a measurable change in protein conformation as demonstrated also in NADP^+^ titration experiments (Figure 2B). The change in FRET (CPR-DA excited at 495 nm) was monitored on mixing oxidized and 2-electron reduced CPR-DA with a saturating concentration (5 mM) of NADP^+^. Example traces are given in Figure S6B–C. The observed changes in emission following mixing with NADP^+^ are similar to those recorded for photo-bleaching. However, we observed small shifts in the absolute magnitude of fluorescence at *t* = 0 for enzyme versus NADP^+^ mixes compared to enzyme versus buffer control mixes. This indicates a loss in the fluorescence signal of a magnitude similar to the titration study (Figure 2B) in the dead time of the stopped-flow instrument, consistent with fast (<5 ms) conformational closure of CPR. Since coenzyme-induced closure of CPR is fast, we infer that any fluorescence changes observed beyond the instrument dead time in reactions of CPR with NADPH would be related to conformational change accompanying chemical (redox) change in the enzyme catalytic cycle.

**Figure 3:**
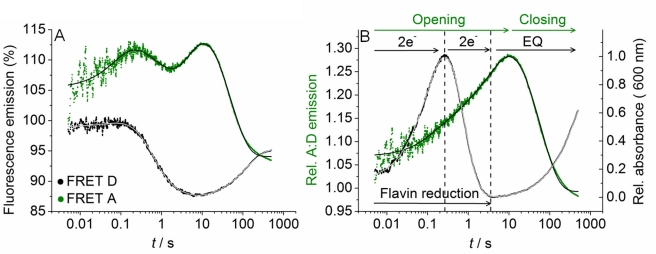
The observed rate of conformational change and flavin reduction in CPR are the same. (A) Example stopped-flow traces of the deconvoluted trace arising from FRET alone for the donor (black) and acceptor (green). Solid lines show the simultaneous fit of both traces to Equation S1. (B) The green line shows the ratio of acceptor to donor emission extracted from the deconvoluted traces (A). The black line shows an example trace for the relative change in CPR absorbance at 600 nm versus a saturating concentration of NADPH (flavin reduction). Solid lines show the fit to Equation S1. The dotted lines show the relative phases of flavin reduction (2e^−^) and establishment of the internal equilibrium (EQ). Conditions: 50 mM potassium phosphate pH 7, 5 mM NADPH at 15°C. Labelled CPR and unlabelled CPR concentrations were ∼0.5 and 40 µM, respectively.

We extracted time-resolved changes in FRET between the D and A fluorophores as flavin reduction proceeds. In this way we assessed relative conformational change associated with flavin reduction in CPR. The time-resolved FRET response is deconvoluted from other contributions to the emission response such as quenching by aromatic residues or FRET involving the flavin moieties in a similar manner as our NADP^+^ titration study (Figure 2B). This is achieved by subtracting the traces for CPR that contained only a single fluorophore (species CPR-D and CPR-A; fluorescence traces shown in Figure S7A and B, trace (i)) from the fluorescence traces for the corresponding fluorophore in a FRET pair (Figure S7B, trace (ii)). The resulting difference traces (Figure 3A) then show the fluorescence emission due to FRET between the extrinsic fluorophores alone. Opposition of the D and A traces was not observed (as expected for FRET data) despite deconvolution of the FRET response, suggesting we recover an approximation of the pure FRET signal. Consequently, we have not calculated detailed distance information from the FRET data, but simply used the FRET signal qualitatively to follow changes in the distribution of CPR conformations in a time-resolved manner. To extract rate constants for the observed changes in the conformational distribution, we simultaneously fit both the donor and acceptor traces in Figure 3A to a multi-exponential expression (Text S1) with linked rate constants for each kinetic phase. This method is robust as the shifting sign of the amplitude for each kinetic phase facilitates good resolution of potentially similar rate constants and small amplitudes. Data fitting is described in detail in Supporting Information (Text S1). The extracted observed rate constants and amplitudes are given in Table S1. These data can be minimally fit to a four exponential function, suggesting there are at least four conformational transitions that occur during flavin reduction by NADPH. The extracted rate constants are essentially the same for each kinetic phase for any of the traces shown in Figure S7 (Table S1). This is consistent with our assertion that the observed changes in fluorescence emission of the fluorophores are due to conformational changes in the enzyme only. That is, the traces give the same rate constants, despite different mechanisms (quenching, FRET, etc.), since the changes in fluorescence emission are caused by the same conformational change as flavin reduction proceeds. Moreover, changes in the FRET signal report specifically on distance changes between C228 and C472/C556 as shown by control experiments in which negligible changes in A∶D ratio were seen with a variant form of CPR containing the C228S mutation (see Text S1 and Figures S8 and S9).

Absorption studies of flavin reduction by NADPH in CPR using stopped-flow methods have previously been reported [12],[24],[31] and can be used to dissect flavin reduction in CPR in detail. These studies indicate that NADPH binds to the FAD domain where it transfers a hydride to the N5 of FAD followed by electron transfer from FAD to FMN to yield a distribution of 2-electron reduced species (FADH^•^ FMNH^•^, FADH_2_ FMN and FAD FMNH_2_). In the absence of an electron acceptor (such as CYP) a second equivalent of NADPH binds to the FAD domain and transfers a hydride to FAD, driving the equilibrium distribution of enzyme states towards the fully (4-electron) reduced species (FADH_2_ FMNH_2_). The observed rate constants for formation of 2-electron (FMNH^•^ FADH^•^) and 4-electron (FMNH_2_ FADH_2_) reduced CPR can be monitored by following the formation and decay of the di-semiquinoid (FMNH^•^ FADH^•^) 2-electron reduced species at 600 nm on mixing with a saturating concentration of NADPH in a stopped-flow instrument [31]. The two exponential phases extracted from these reaction traces correspond broadly to the observed rate constants for 2-electron and 4-electron reduction (termed *k*_1_ and *k*_2_, respectively). CPR-DA reacts with NADPH in a similar way, and the kinetics of absorption change at 600 nm for the two exponential phases are identified as “flavin reduction” (black trace) in Figure 3B. Further, there is a very slow phase (increase in 600 nm absorbance; represented as “EQ” in Figure 3B) observed after 4-electron reduction. In wild-type CPR, this phase has been attributed previously to the formation of an internal equilibrium between redox states in the absence of an electron acceptor [31]. This slow adjustment to the final equilibrium of redox states is retained in CPR-DA. In the present study, we focus on the chemical steps, *k*_1_ and *k*_2_. Figure 3B shows a typical reaction trace for flavin reduction by NADPH in CPR monitored at 600 nm. The data are fit to a four exponential function (Text S1, Equation S1) accounting for all the observed absorption changes discussed above. The rate constants for *k*_1_ and *k*_2_ at 25°C are given in Table 1, extracted as *k*_obs1_ = 16.2±0.2 s^−1^ and *k*_obs2_ = 4.0±0.1 s^−1^. Table 1 also shows the observed rate constants for the first two kinetic phases extracted from the fluorescence data that represent conformational change (Figure 3A). The rate constants extracted from the fluorescence data (*k*_obs1_ = 24.5±1.0 s^−1^ and *k*_obs2_ = 4.2±0.1 s^−1^) are similar to the observed rate constants for flavin reduction, suggesting that flavin reduction and conformational change are linked in CPR.

**Table 1:** Observed rate constants for 2- and 4-electron reduction of CPR monitored as a change in 600 nm absorbance (flavin reduction) and fluorescence emission of extrinsic fluorophores (domain motion).

Reduction Level of CPR	Observed Rate Constants and Enthalphy Changes for 2- and 4-Electron Reduction	Flavin Reduction	Domain Motion
2e^−^	*k*_obs1_ (s^−1^)^a^	16.2±0.2	24.5±1.0
	Δ*H^‡^*_1_ (kJ mol^−1^)	61.1±0.3	62.4±3.4
4e^−^	*k*_obs2_ (s^−1^)^a^	4.0±0.1	4.2±0.1
	Δ*H^‡^*_2_ (kJ mol^−1^)	64.1±0.2	67.6±2.8

a Measurement at 25°C.

We now correlate the observed changes in flavin redox state with the conformational changes extracted from our fluorescence data. Figure 3B shows the ratio of acceptor to donor (A∶D) emission extracted from the stopped-flow traces shown in Figure 3A, effectively describing the trend in FRET efficiency during flavin reduction by NADPH. These data clearly show that the FRET signal is increased (i.e., more “open” conformations are populated) as CPR is sequentially reduced to the 2-electron and then 4-electron levels (indicated by the time-resolved absorption measurements at 600 nm; Figure 3B). Further, following flavin reduction we observed a gradual closing of CPR (reduced A∶D emission ratio) over prolonged time periods (10 to 1,000 s) as CPR relaxes to the final equilibrium position. These FRET data indicate, therefore, that conformational closure is a key part of this long-time base equilibration of the reduced enzyme species and that the more open state is a metastable form of reduced CPR. The fluorescence and absorption changes shown in Figure 3B occur over very similar timescales (0–10 s), suggesting that domain motion is linked to redox chemistry. The potential for direct coupling of redox change with conformational opening of CPR is addressed below.

### CPR Exhibits Redox-State Dependent Conformational Change

**Figure 4:**
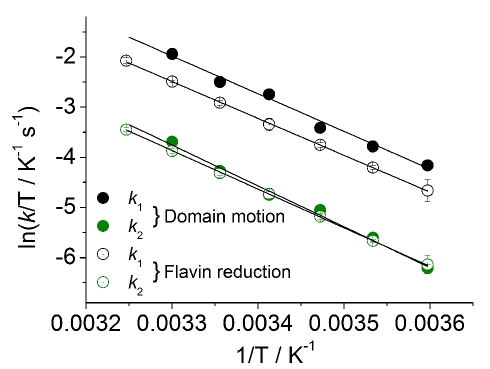
The temperature dependence of the observed rate of flavin reduction and domain motion in CPR are the same. Temperature dependence data for conformational change (filled circles) and flavin reduction (open circles) are extracted from stopped-flow fluorescence and absorbance measurements, respectively. Data are shown for both 2-electron (black, *k*_1_) and 4-electron (green, *k*_2_) reduction. Black lines show the fit to the Eyring equation and extracted parameters are reported in Table 1. Conditions: 50 mM potassium phosphate pH 7. Labelled CPR and unlabelled CPR concentrations were ∼0.5 and 40 µM, respectively.

Strong evidence for the coupling of conformational change with enzyme chemistry would arise if the energetic barriers for electron transfer and motion are shown to be equivalent. This was addressed experimentally by monitoring the temperature dependence of the rate of flavin reduction (absorption change at 600 nm, reporting on *k*_1_ and *k*_2_) and associated conformational change (from time-resolved fluorescence data). The temperature dependence of the rate constants *k*_1_ and *k*_2_ for structural change and flavin reduction is shown in Figure 4. The Eyring plots for the fluorescence data are linear (Figure 4), suggesting that the first two exponential phases each report on rate constants for a single process (i.e., structural change). The values of Δ*H* ^‡^ for flavin reduction and conformational change are given in Table 1 and are similar for both kinetic phases (*k*_1_ and *k*_2_). That Δ*H* ^‡^ is very similar for both flavin reduction and structural change for both exponential phases (2-electron and 4-electron reduction) suggests a tight coupling of the reaction chemistry with the observed structural transitions.

**Figure 5:**
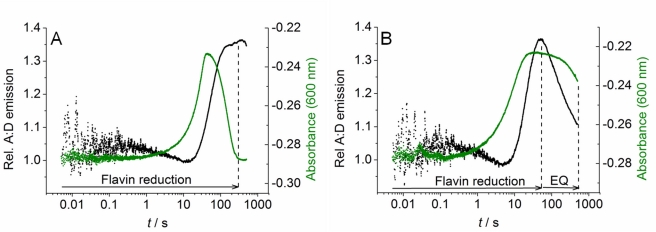
Redox state dependent conformational changes in CPR. Example stopped-flow traces for flavin reduction (green) and A∶D emission (black) with sodium dithionite (A) or NADP^+^+dithionite (B). The dotted lines show the approximate end points of the 4-electron reduced oxidation state and the slow equilibrium of redox states (EQ). Conditions: 50 mM potassium phosphate pH 7, 5 mM NADP^+^ at 20°C. Labelled CPR and unlabelled CPR concentrations were ∼0.5 and 40 µM, respectively.

The kinetics and energetics of flavin reduction and conformational change are consistent with the two processes being tightly coupled. These data might suggest that flavin reduction is responsible for conformational opening of CPR or that conformational change induces electron transfer associated with flavin reduction. The analysis, however, is complicated by the opposing effects of coenzyme binding, which in the absence of redox chemistry is known to effect closure of CPR (Figure 2B). We therefore conducted stopped-flow measurements in which CPR-DA was mixed with the chemical reductant, sodium dithionite, to investigate the effects of redox change in the absence of coenzyme binding. A typical trace from these experiments is shown in Figure 5A. As with NADPH reduction, when dithionite is used to reduce the flavin centers the appearance and subsequent disappearance of the flavin semiquinones is observed at 600 nm corresponding to formation of the 2-electron and 4-electron reduced species of CPR. The individual exponential components corresponding to 2-electron and 4-electron reduction are less well defined, which complicates data analysis, but approximate rate constants can be obtained. The observed rate constant for flavin reduction is far slower, with dithionite being ∼0.05 s^−1^ and ∼0.04 s^−1^, compared to NADPH, ∼19 s^−1^ and ∼2.5 s^−1^, at 20°C for 2- and 4-electron reduction, respectively. The change in FRET efficiency for CPR-DA was also monitored as flavin reduction proceeds (Figure 5A). Individual fluorescence traces used to calculate the FRET response are shown in Figure S11A,B. The change in FRET efficiency can be adequately fit to a two-exponential function (Text S1, Equation S1) with observed rate constants of ∼0.05 s^−1^ and ∼0.03 s^−1^ for the first and second kinetic phases, respectively. In general, there is a large increase in A∶D (CPR opening) as flavin reduction proceeds. We note that the first exponential phase shows a slight decrease in A∶D, though this is on a faster timescale than either 2- or 4-electron reduction (Figure 5A). Therefore, as seen with NADPH, the rate of flavin reduction by dithionite correlates with the observed rate of conformational change (Figure 5) and reduction to the 2-electron and 4-electron levels is accompanied by an opening of CPR. These data indicate that reduction of the flavin cofactors alone is sufficient to induce conformational opening of CPR. Further, these data are consistent with our temperature dependence data from which we inferred a tight coupling of the conformational transition with the flavin redox state.

We also examined the effects of coenzyme binding on the rate of flavin reduction and conformational opening during enzyme reduction with dithionite. This is achieved by mixing CPR-DA that had been pre-incubated with a saturating concentration of NADP^+^ (5 mM) with dithionite. There was no evidence for reduction of NADP^+^ to NADPH (monitored by absorption changes at 340 nm) by dithionite over the timescale of the study. In the presence of NADP^+^, the rate constant for flavin reduction by dithionite (2-electron reduction) increases approximately 2-fold (∼0.01 s^−1^; Figure 5B) compared with reactions performed in the absence of NADP^+^. The conversion of 2-electron reduced CPR to the 4-electron reduced species is not well-resolved, due likely to overlap with the slow kinetic phase(s) involved in establishing the final equilibrium of redox states (EQ) (Figure 5B). The corresponding change in FRET efficiency is shown in Figure 5B with the individual fluorescence trace shown in Figure S11C and S11D. As with dithionite alone, there is a significant increase in the A∶D ratio corresponding to CPR opening and this occurs on a similar timescale to flavin reduction (approximate rate constant ∼0.08 s^−1^).

The effect of NADP^+^ is therefore to accelerate (approximately 2-fold) flavin reduction and the associated conformational opening of CPR with dithionite as reductant. Further, after the opening of CPR with NADP^+^ bound there is a subsequent decrease in A∶D reflecting CPR closure as the reduced enzyme relaxes to the final equilibrium (EQ) state (Figure 5B). On the timescale of our measurements we do not observe the establishment of this equilibrium in dithionite studies performed in the absence of NADP^+^ (Figure 5A). We find therefore that NADP^+^ not only increases the observed rate of flavin reduction, but also increases the observed rate of EQ formation. This is consistent with previous t-jump studies of interflavin electron transfer in di-semiquinoid human CPR, where the observed rate of electron transfer (55 s^−1^) is increased 5-fold on adding NADP^+^ compared to reactions performed in the absence of nicotinamide coenzyme [20]. The precise reasons for accelerated flavin reduction in the presence of NADP^+^ are unclear. However, cofactor binding likely induces a shift in the equilibrium distribution of enzyme forms towards a more closed conformation (Figure 2B). We suggest that internal electron exchange between the flavin cofactors is enhanced due to a minimized cofactor separation induced by NADP^+^ binding. Clearly, once CPR is reduced by dithionite the distribution then adjusts first to the metastable open conformations (0–50 s) and then relaxes to the more closed EQ conformations (>50 s) (Figure 5B).

### An Integrated Model for Dynamics and Enzyme Chemistry in CPR

**Figure 6:**
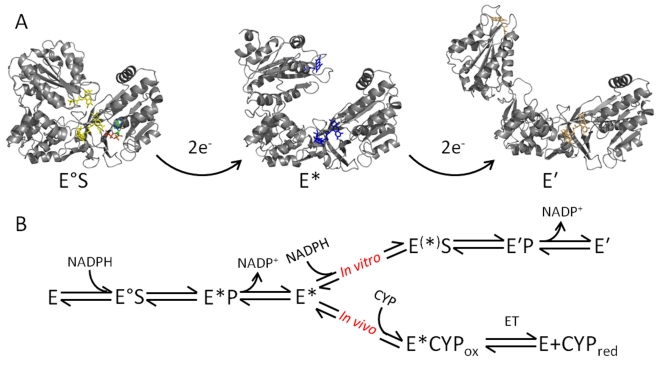
Integrated model of dynamics and chemistry in CPR in a proposed 2,4 reaction cycle as observed in vitro. The structures shown are taken from homology models of available rat CPR X-ray crystal structures and are used to exemplify particular conformational changes. (A) CPR undergoes large scale domain motion subsequent to flavin reduction. There is a detectable conformational change associated with the 2- and 4-electron reduced state of CPR, giving the species E/E° (oxidized, yellow flavin), E* (2-electron reduced, blue flavin), and E′ (4-electron reduced, orange flavin). Binding of NADPH (green sticks) to oxidized (E) or 2-electron reduced (E*) CPR causes a relative closing to form E°S and E^(^*^)^S, respectively. (B) A model for directional transfer of electrons in vitro and in vivo. The 2-electron reduced form of CPR (E*) is capable of being further reduced (E′) in vitro. However, in vivo electrons are transferred from the open CPR (E*) to the heme of a CYP partner. A similar scheme for a 1,3 catalytic cycle is possible in vivo in which conversion of enzyme species E° to E* represents reduction of 1-electron reduced CPR to 3-electron reduced CPR (see text for details).

We have monitored two separate conformational transitions in CPR, namely opening and closing, which correspond to increased and decreased separation of the FMN and FAD cofactors, respectively. Opening is driven by reduction of the flavin cofactors and closure by coenzyme binding (Figure 6A). We now develop an integrated model for CPR action that incorporates these conformational transitions (Figure 6). In this model, domain motions driven by flavin reduction are crucial in mediating electron transfer to CYPs. The open conformations expose residues required for CYP interaction that are occluded in the closed conformation [25]. The open state signals that CPR is “ready and waiting” to transfer electrons to CYP partners in the microsomal membranes. Should productive interaction with CYP partners not occur, subsequent closure of CPR (formation of EQ) may offer some protection of the reducing equivalents in the flavin cofactors by suppressing their adventitious transfer (e.g., to molecular oxygen). The open conformation of CPR is not appropriate for “electron loading” from the reducing coenzyme NADPH. Rapid equilibration of electrons across the flavin centers is required to generate 2- and 4-electron reduced CPR from the oxidized form. Therefore, “closure” of the oxidized form of CPR is induced on nicotinamide coenzyme binding to facilitate efficient loading with reducing equivalents prior to redox-driven opening of the structure. We note that there is evidence for both 2, 4 and 1, 3 electron cycling in CPR [32],[33], and we propose a similar mechanism of opening/closing driven by the flavin redox state can occur in either case. However, in vivo the need for a second hydride transfer from NADPH may be less important (Figure 6B). The redox and ligand-bound forms of CPR therefore drive the re-distribution of CPR conformations across the associated energy landscape to more open or closed forms of CPR. These different conformations direct downstream interaction of CPR with CYP partners and facilitate directional transfer of reducing equivalents for CYP-mediated catalysis. Such motion therefore drives the vectorial transfer of electrons from NADPH to CYP to catalyze the wide range of mono-oxygenation reactions in the endoplasmic reticulum.

It is important to distinguish between the relatively large-scale redox-coupled and ligand-coupled motions discussed above and other stochastic motions that can limit the rate of electron transfer. Our model therefore also recognizes that smaller-scale motions can also limit electron transfer, either between flavin cofactors (in the closed state) or to CYP enzymes (in the open state). Localized searches for productive reaction geometries are common in biological electron transfer reactions and these are often responsible for the slower observed rates of electron transfer compared to those predicted for “pure” (nonadiabatic) electron transfers on the basis of distance criteria alone [23],[34]. Indeed, our temperature-dependence studies indicate that the reaction cannot be modeled using the Marcus nonadiabatic formalism for electron transfer (see Text S1 for details; Figure S10 and Table S2). As such, Figure 6 should not be taken to imply that single discrete open and closed conformations of CPR exist under a defined ligand-bound or redox form of the enzyme. Rather, an ensemble of conformations exist (as indicated by PELDOR studies of different liganded forms of di-semiquinoid CPR [12]) and that redox change and ligand binding drive the equilibrium distribution towards more open or closed states, respectively. We suggest that localized searches for reactive electron transfer geometries could be rate limiting for interflavin electron transfer in CPR, consistent with the slow observed rates of electron transfer in t-jump [20] and laser flash photolysis experiments [20],[35].

### Conclusion

The use of time-resolved FRET in conjunction with stopped-flow absorption analysis of the CPR catalytic cycle has enabled us to present a dynamic model of catalysis in which redox change and ligand binding drive large-scale redox domain motion. By coupling conformational change to redox change and ligand binding, CPR optimizes internal electron movements between the flavin cofactors and signals “open and ready” conformations to partner CYP P450 enzymes. This linking of motion with enzyme chemistry enables fine control of vectorial electron transfer along the NADPH→FAD→FMN→heme (CYP) chain that supports all P450-mediated catalysis in the microsome. Given the structural similarity of CPR with other major mammalian diflavin oxidoreductases, including the isoforms of nitric oxide synthase, we anticipate CPR will be a prototype for similar coupling of reaction chemistry, ligand binding, and motions in biology.

## Materials and Methods

### Homology Models of Human CPR

The human CPR structures were modeled using SwissModel from the rat CPR crystal structures. For the closed and intermediate forms, the sequence was simply fit to the crystal structure (1AMO_A and 3ES9_C, respectively, with 94% and 93% sequence identity). Loops not present in the crystal structures were modeled using the built-in loop database (closed form: residue 235–241 and 499–505; intermediate form: 235–238 and 499–504). For the most open form, a significant portion of the FMN domain is missing in the crystal structure (3ES9_B). This portion was modeled by aligning the FMN domain from the intermediate form onto the existing coordinates of the crystal structure with the loop connecting the two domains modeled using the built-in loop database.

### Extrinsic Fluorophore Labeling of CPR

Human CPR was expressed and purified essentially as described previously [31]. Labeling of CPR with extrinsic fluorophores was achieved by incubating CPR in 50 mM potassium phosphate, pH 7 at <20°C in an anaerobic glove box (Belle Technology) with either Alexa 488 C5-maleimide (Molecular Probes) or Cy 5 mono-maleimide (GE Healthcare). To achieve a 1∶1 ratio of Alexa 488 and Cy 5 bound to CPR, incubation was with 1 mM and 5 mM of the fluorophores, respectively. Non-reacted fluorophore was separated from the sample by running through a desalting column equilibrated with 50 mM potassium phosphate, pH 7. Details of mass spectral analysis are given in Supporting Information (Text S1). Unless otherwise stated, CPR was fully oxidized prior to all experiments by adding a few grains of potassium ferricyanide and elution through a desalting column as above. 2-electron reduced CPR was formed by reaction with an equimolar concentration of NADPH (Melford), and elution through a desalting column under anaerobic conditions.

### Static Fluorescence and Absorbance Measurements

Fluorescence emission spectra were monitored on a Varian Cary Eclipse fluorescence spectrophotometer (Varian Inc., Palo Alto, CA, USA). Multiple wavelength absorbance spectra were monitored on a Varian Cary 50 Bio UV/Vis spectrophotometer. Specific experimental conditions are given in the main text. All experiments were performed in 50 mM potassium phosphate, pH 7. The saturation constant, *K*_S_, was extracted by fitting concentration dependence data to a weak binding function (Equation 1):
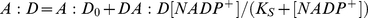
(1)


### Stopped-Flow Studies and Data Fitting

To prevent oxidase activity of CPR, all kinetic studies were performed under strict anaerobic conditions within a glove box (Belle Technology; <5 ppm O_2_) using a Hi-Tech Scientific (TgK Scientific, Bradford on Avon, UK) stopped-flow spectrophotometer housed inside the glove box. Spectral changes accompanying flavin reduction and flavin di-semiquinone formation/decay were monitored at 456 nm and 600 nm, respectively. For reduction with sodium dithionite, the same solution of dithionite was used for all experiments within 6 h. Fluorescence emission changes associated with donor emission were monitored using a 550 nm short wave pass optical filter. Fluorescence emission changes associated with acceptor emission were monitored using a 650 nm long wave pass optical filter. For the given reaction conditions, no bleed through of fluorescence was observed. Data were collected over a log timebase (15 decades, 3,000 data points total). Typically 3–5 measurements were taken for each reaction condition. Fitting of reaction traces is described in detail in Supporting Information (Text S1).

## Supporting Information

Figure S1 Absorbance spectra of CPR-DA. Two molar equivalents of the donor and acceptor probes are bound at a 1∶1 ratio. Extinction coefficients are: CPR (456 nm) ε = 22 mM^−1^ cm^−1^, Alexa 488 (495 nm) ε = 72 mM^−1^ cm^−1^, and Cy 5 (655 nm) ε = 250 mM^−1^ cm^−1^. The flavin absorbance is overlapped by Alexa 488 absorbance. Flavin absorbance is calculated based on the known absorbance ratio 280 nm∶456 nm. Conditions: 50 mM potassium phosphate pH 7, 20°C, 0.6 µM CPR-DA.
(TIF)

Figure S2 Mass spectral analysis of fluorophore Alexa 488 labeled CPR. (A) HPLC trace of labeled (red) and unlabelled (black) CPR monitoring protein absorbance at 215 nm (top panel) and Alexa 488 absorbance at 475 nm (bottom panel). Black dashed lines show fractions which were taken for MS/MS analysis. (B) Example spectra showing labeled peptides for positions C228 (top panel), C472 (middle panel), and C566 (bottom panel).
(TIF)

Figure S3 Emission of the Cy 5 dye and quenching by CPR. (A) The black line shows the Cy 5 dye excited at 655 nm, and the red line shows CPR-A excited at 655 nm. The ratio of peak integrals for Cy 5 and CPR-A excited at 655 nm is 0.23. This is then the relative quenching of the dye associated with binding to CPR. (B) Excitation of CPR-A (red line) at 495 nm gives rise to a small emission peak at ∼670 nm. The magnitude of this emission is ∼3% of that attributable to Acceptor emission arising from FRET (black line). Conditions: 0.5 µM Cy 5 and CPR-A, 50 mM potassium phosphate, pH 7 at 20°C.
(TIF)

Figure S4 Changes in fluorescence report on conformational change only. (A) Fluorescence emission spectra of CPR-D (red), CPR-DA (black), and an equimolar mix of CPR-D and CPR-A (blue) excited at 495 nm. *Inset*, zoomed in view of emission from Cy 5. (B) Flavin fluorescence emission of oxidized CPR (black), oxidized CPR-A (red), and Cy 5 normalized for relative quenching by the protein (blue) excited at 456 nm. Conditions: 50 mM potassium phosphate pH 7, 25°C. CPR and fluorophore concentrations were 0.35 µM.
(TIF)

Figure S5 Titration of NADP^+^ against (A) CPR-D (black, Ex 495 nm, Em <550 nm) and CPR-A (green, Ex 655 nm, Em >650 nm) and (B) CPR-DA (Ex 495 nm, black Em <550 nm, green Em >650 nm). The solid lines show the fit to Equation 1. The concentration-dependencies in (B) are adjusted for the relative change in emission of the respective donor/acceptor only emission (A) as described in Materials and Methods. Conditions: 0.4 µM CPR-DA, CPR-D and CPR-A, 50 mM potassium phosphate, pH 7 at 20°C.
(TIF)

Figure S6 Transient state kinetics of donor and acceptor fluorophore emission on mixing with NADP^+^. Panel (A) shows CPR-D and CPR-A emission versus buffer. Panel (B) shows CPR-DA emission versus saturating NADP^+^ or (C) 2e− reduced CPR-DA versus saturating NADP^+^. These data are not corrected for the variation in emission due to the individual fluorophores (CPR-D and CPR-A) as the traces are essentially identical, showing only changes associated with photo-bleaching of the fluorophores. The emission of the traces has been normalized in each case to 100% at t = 0, but see Materials and Methods for more details. Conditions: 0.4 µM CPR-DA, 50 mM potassium phosphate, pH 7 at 25°C and 5 mM NADP^+^ (B/C).
(TIF)

Figure S7 Example stopped-flow traces and residuals for donor (A) and acceptor (B) emission upon flavin reduction. The emission is given as percentage change, where t = 0 is 100%; see Materials and Methods for details. Trace (i) shows the emission from the singly labeled enzyme, CPR-D or CPR-A, excited at 495 nm and 655 nm, respectively. Trace (ii) shows the emission from CPR-DA exited at 495 nm. Trace (iii) shows the subtraction of trace (i) from trace (ii) to give the change due in emission due to FRET only. Residuals for each fit (A and B) are shown below the respective panel and have the corresponding color. The residuals are essentially randomly distributed over the time range. Conditions: 0.4 µM CPR-DA, 50 mM potassium phosphate, pH 7 at 25°C and 5 mM NADPH.
(TIF)

Figure S8 Transient state kinetics of CPR reduction and conformational change in the C228S CPR variant. (A) Trace showing absorbance change attributable to flavin reduction, reflecting the 2-electron reduced (increase in absorbance) and 4-electron reduced (decrease in absorbance) states. (B) Example traces of the deconvoluted donor and acceptor emission as in Figure S7. (C) The change in A∶D emission ratio extracted from the data in panel (B) on the timescale of flavin reduction. Conditions: 0.4 µM C228S CPR-DA, 50 mM potassium phosphate, pH 7 at 15°C and 5 mM NADPH.
(TIF)

Figure S9 Deconvolution of the FRET response arising from FAD inter-domain motion upon flavin reduction. The A∶D emission ratio for wild-type CPR (i) is shown with the C228S CPR variant (ii) on an extended time base. Deconvolution of the C228S CPR response from that of the wild type enzyme gives rise to trace (iii).
(TIF)

Figure S10 Temperature-dependence of the observed rate of flavin reduction (A) and domain motion (B) fit to the Marcus equation (Text S1, Equation S2). The values for *k*_obs_ were extracted as described in Materials and Methods and are only given for the first two kinetic phases. The resulting parameters from fitting to the Marcus equation are given below (Table S2). Conditions: 50 mM potassium phosphate pH 7, 0.5 µM CPR-DA and 5 mM NADPH.
(TIF)

Figure S11 Example stopped-flow transients donor (A/C) and acceptor (B/D) emission upon flavin reduction with sodium dithionite in the absence (A/B) or presence (C/D) of bound NADP^+^. The emission is given as percentage change, where t = 0 is 100%; see Materials and Methods for details. Transient (i) shows the emission from the singly labeled enzyme (CPR-D or CPR-A). Transient (ii) shows the emission from CPR-DA exited at 495 nm. Transient (iii) shows the subtraction of transient (i) from transient (ii) to give the change due in emission due to FRET only. Conditions: 0.4 µM CPR-DA, 50 mM potassium phosphate, pH 7 at 20°C and 5 mM NADPH.
(TIF)

Table S1 Donor and Acceptor emission extracted from the trace in Figure S7 and Figure 3A.
(DOC)

Table S2 Parameters derived from fitting the Marcus equation to the temperature-dependence of the rate constants in Figure S10.
(DOC)

Text S1 Supplementary text to the Materials and Methods, and Results and Discussion sections.
(DOC)

## Abbreviations

CPR: cytochrome P450 reductase
CYP: cytochrome P450
FAD: flavin adenine dinucleotide
FMN: flavin mononucleotide
FRET: fluorescence resonance energy transfer
NADPH: nicotinamide adenine dinucleotide phosphate
PELDOR: pulsed electron electron double resonance
