# Supplementary figures and images for: Coupled Motions Direct Electrons along Human Microsomal P450 Chains
Source: PLoS Biol. 2011 Dec 20;9(12):e1001222. doi: 10.1371/journal.pbio.1001222 (PMC3243717; doi:10.1371/journal.pbio.1001222)

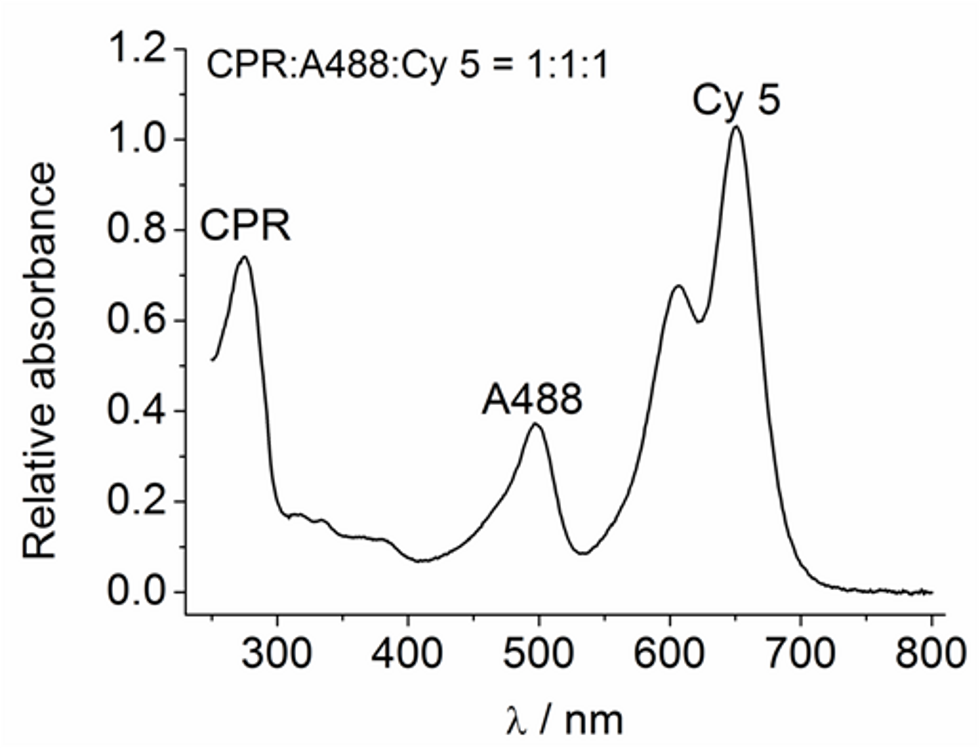

Supplement: Figure S1 — Absorbance spectra of CPR-DA. Two molar equivalents of the donor and acceptor probes are bound at a 1∶1 ratio. Extinction coefficients are: CPR (456 nm) ε = 22 mM−1 cm−1, Alexa 488 (495 nm) ε = 72 mM−1 cm−1, and Cy 5 (655 nm) ε = 250 mM−1 cm−1. The flavin absorbance is overlapped by Alexa 488 absorbance. Flavin absorbance is calculated based on the known absorbance ratio 280 nm∶456 nm. Conditions: 50 mM potassium phosphate pH 7, 20°C, 0.6 µM CPR-DA. (TIF) [file pbio.1001222.s001.tif]

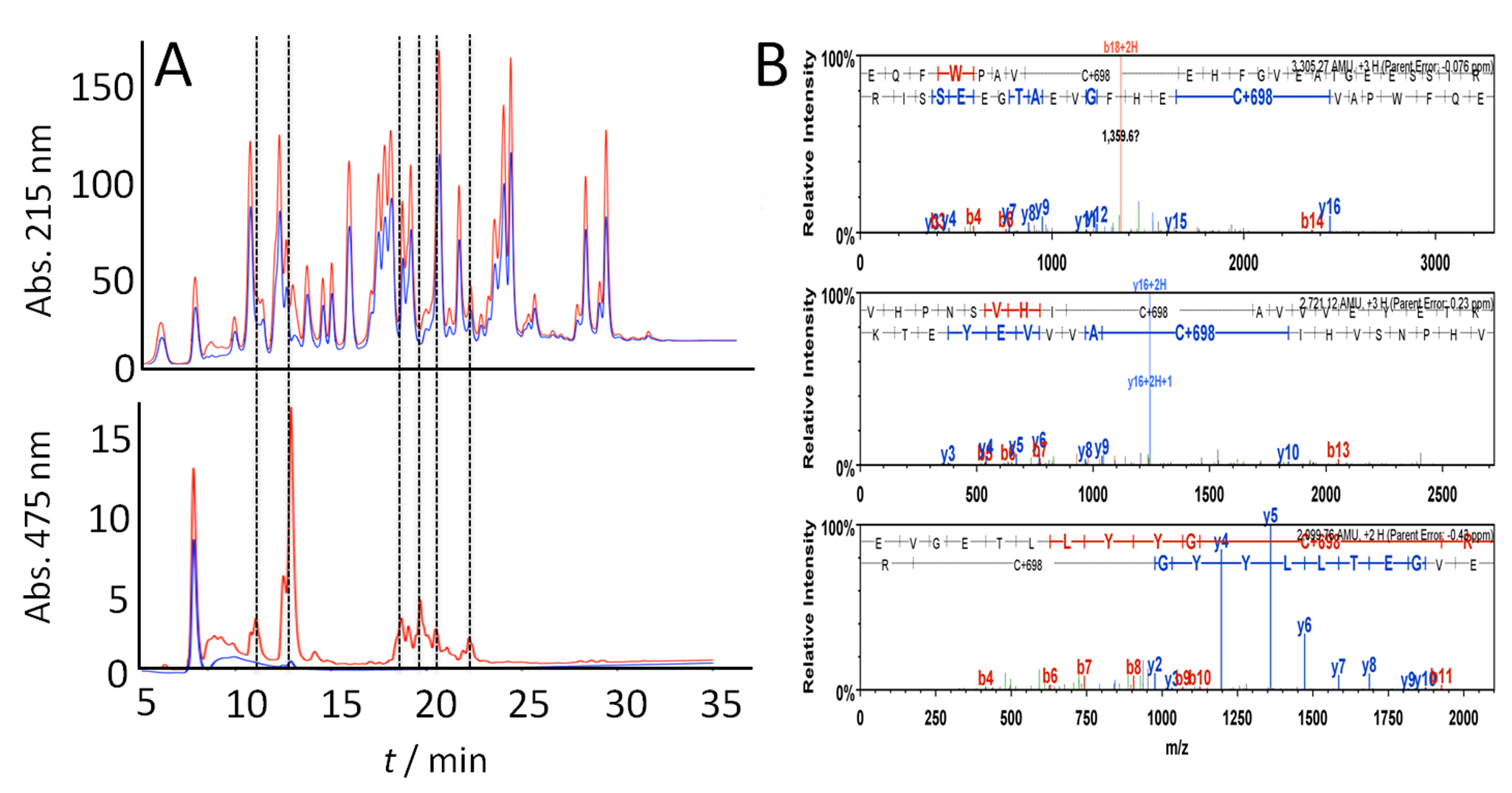

Supplement: Figure S2 — Mass spectral analysis of fluorophore Alexa 488 labeled CPR. (A) HPLC trace of labeled (red) and unlabelled (black) CPR monitoring protein absorbance at 215 nm (top panel) and Alexa 488 absorbance at 475 nm (bottom panel). Black dashed lines show fractions which were taken for MS/MS analysis. (B) Example spectra showing labeled peptides for positions C228 (top panel), C472 (middle panel), and C566 (bottom panel). (TIF) [file pbio.1001222.s002.tif]

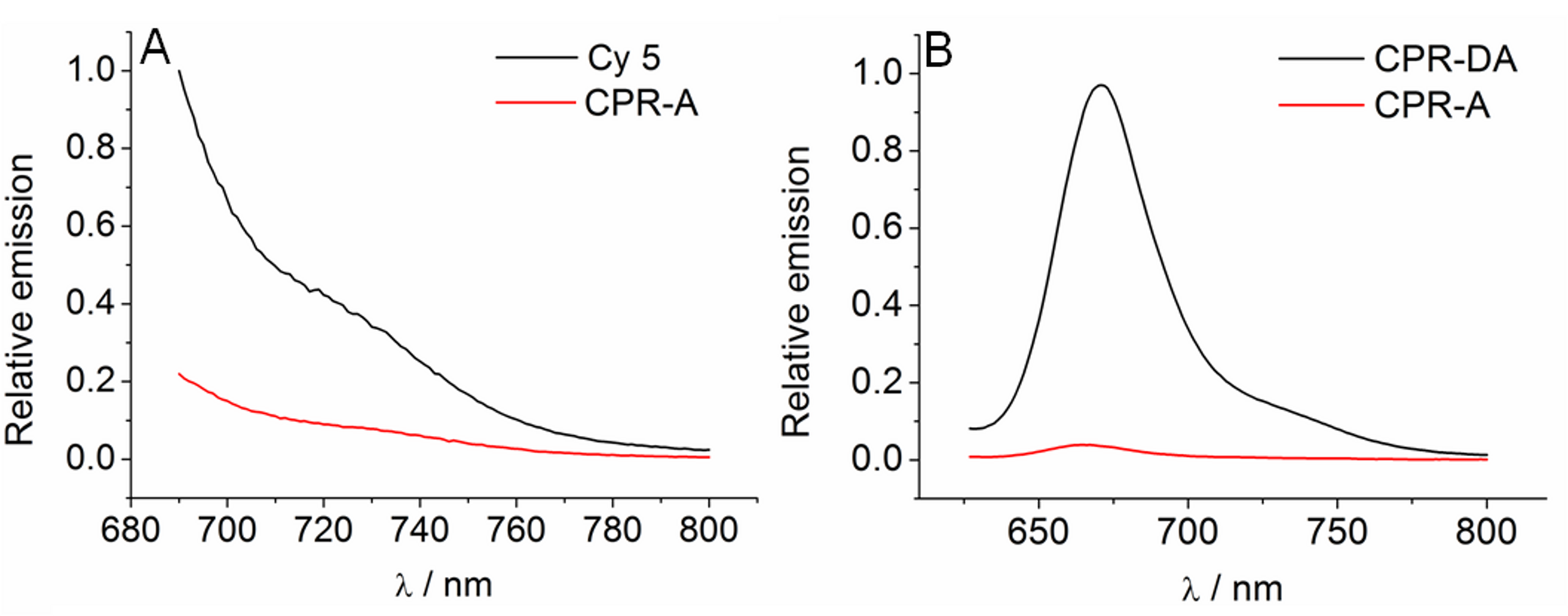

Supplement: Figure S3 — Emission of the Cy 5 dye and quenching by CPR. (A) The black line shows the Cy 5 dye excited at 655 nm, and the red line shows CPR-A excited at 655 nm. The ratio of peak integrals for Cy 5 and CPR-A excited at 655 nm is 0.23. This is then the relative quenching of the dye associated with binding to CPR. (B) Excitation of CPR-A (red line) at 495 nm gives rise to a small emission peak at ∼670 nm. The magnitude of this emission is ∼3% of that attributable to Acceptor emission arising from FRET (black line). Conditions: 0.5 µM Cy 5 and CPR-A, 50 mM potassium phosphate, pH 7 at 20°C. (TIF) [file pbio.1001222.s003.tif]

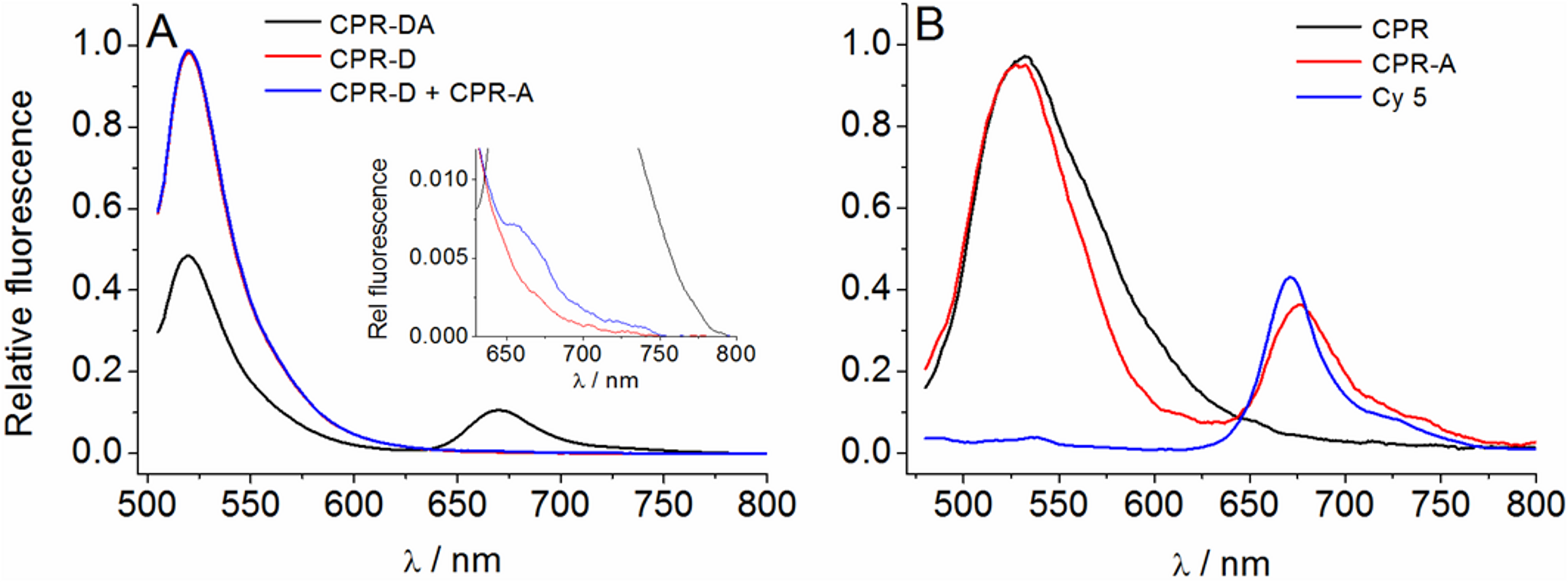

Supplement: Figure S4 — Changes in fluorescence report on conformational change only. (A) Fluorescence emission spectra of CPR-D (red), CPR-DA (black), and an equimolar mix of CPR-D and CPR-A (blue) excited at 495 nm. Inset, zoomed in view of emission from Cy 5. (B) Flavin fluorescence emission of oxidized CPR (black), oxidized CPR-A (red), and Cy 5 normalized for relative quenching by the protein (blue) excited at 456 nm. Conditions: 50 mM potassium phosphate pH 7, 25°C. CPR and fluorophore concentrations were 0.35 µM. (TIF) [file pbio.1001222.s004.tif]

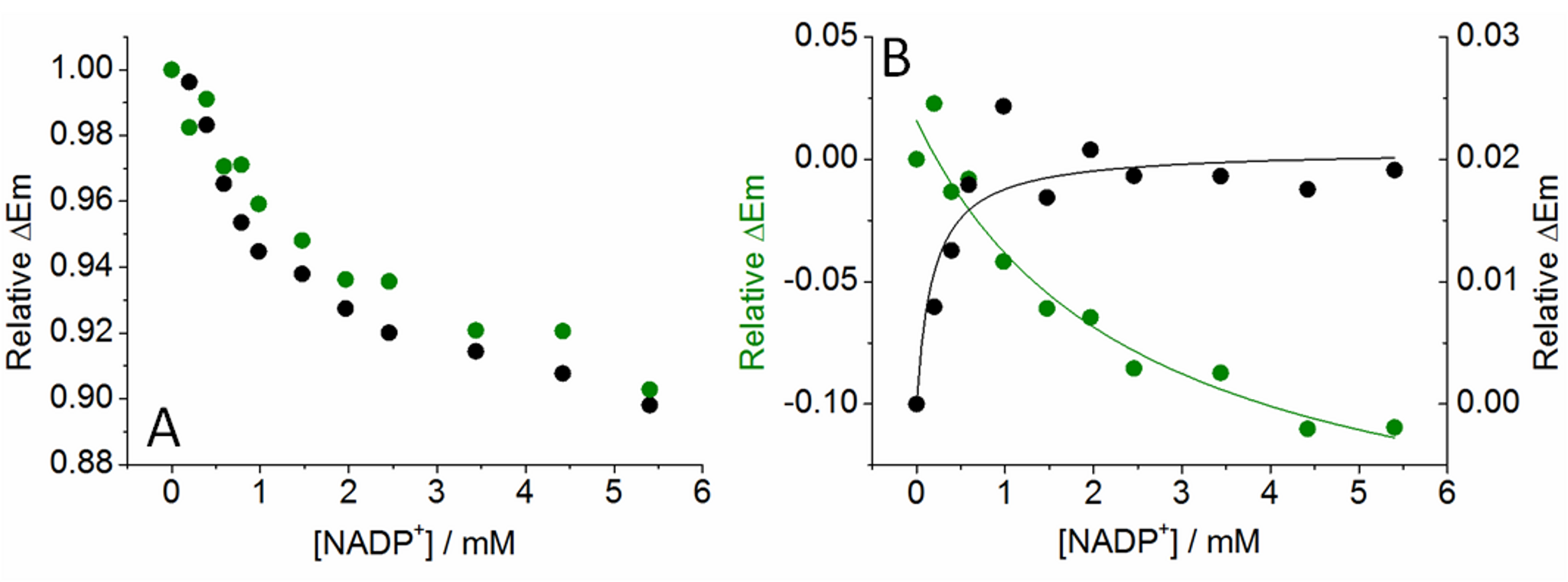

Supplement: Figure S5 — Titration of NADP+ against (A) CPR-D (black, Ex 495 nm, Em <550 nm) and CPR-A (green, Ex 655 nm, Em >650 nm) and (B) CPR-DA (Ex 495 nm, black Em <550 nm, green Em >650 nm). The solid lines show the fit to Equation 1. The concentration-dependencies in (B) are adjusted for the relative change in emission of the respective donor/acceptor only emission (A) as described in Materials and Methods. Conditions: 0.4 µM CPR-DA, CPR-D and CPR-A, 50 mM potassium phosphate, pH 7 at 20°C. (TIF) [file pbio.1001222.s005.tif]

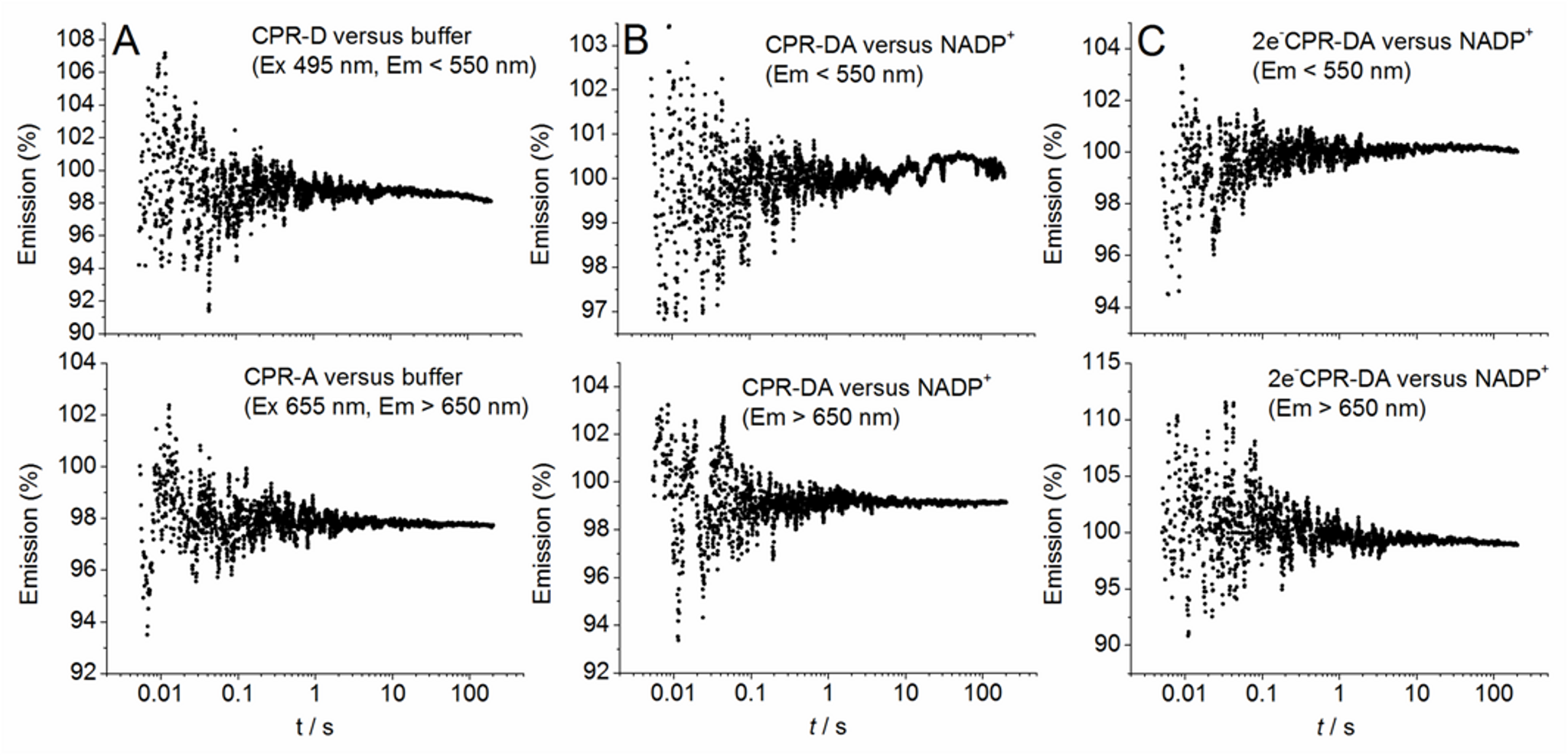

Supplement: Figure S6 — Transient state kinetics of donor and acceptor fluorophore emission on mixing with NADP+. Panel (A) shows CPR-D and CPR-A emission versus buffer. Panel (B) shows CPR-DA emission versus saturating NADP+ or (C) 2e− reduced CPR-DA versus saturating NADP+. These data are not corrected for the variation in emission due to the individual fluorophores (CPR-D and CPR-A) as the traces are essentially identical, showing only changes associated with photo-bleaching of the fluorophores. The emission of the traces has been normalized in each case to 100% at t = 0, but see Materials and Methods for more details. Conditions: 0.4 µM CPR-DA, 50 mM potassium phosphate, pH 7 at 25°C and 5 mM NADP+ (B/C). (TIF) [file pbio.1001222.s006.tif]

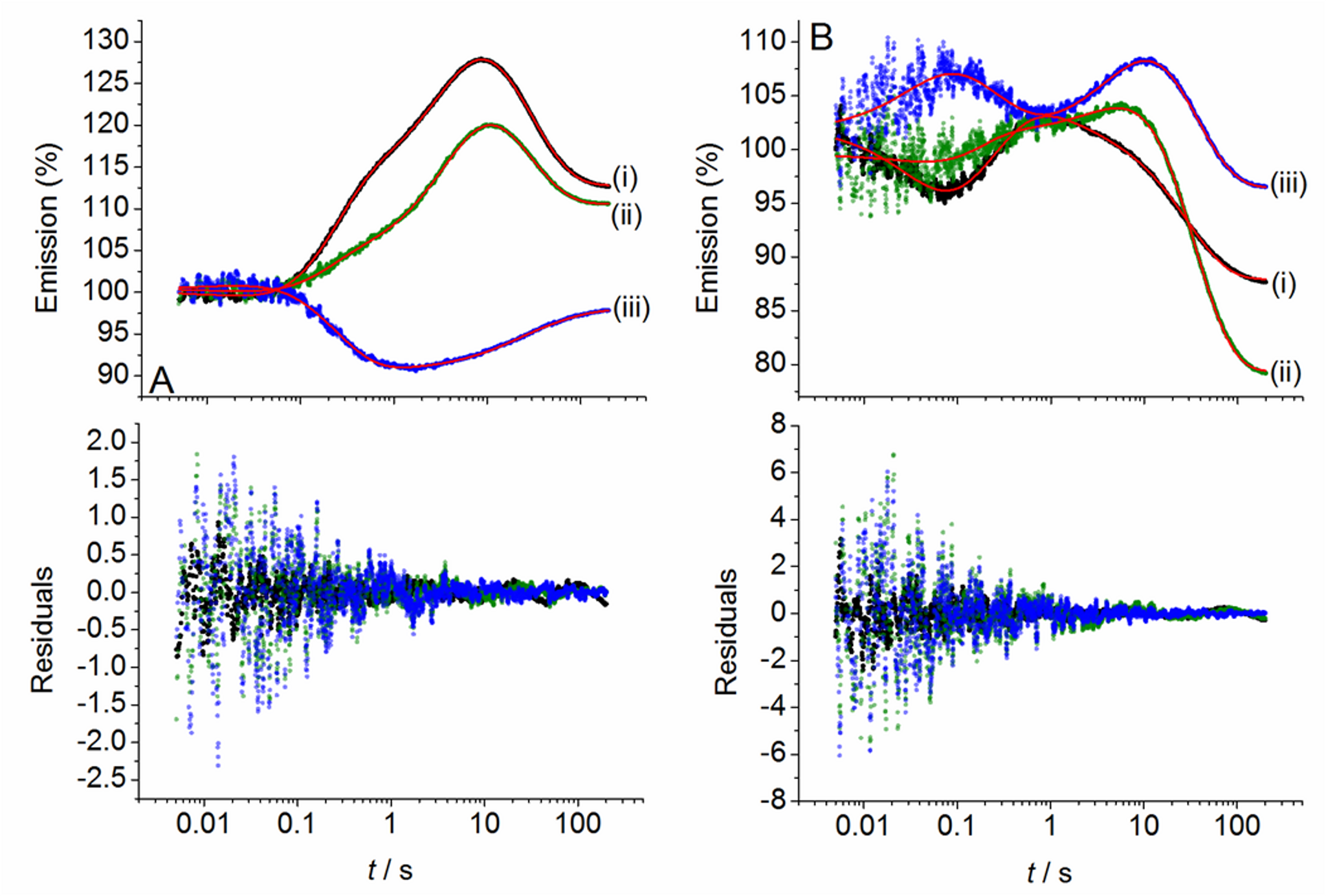

Supplement: Figure S7 — Example stopped-flow traces and residuals for donor (A) and acceptor (B) emission upon flavin reduction. The emission is given as percentage change, where t = 0 is 100%; see Materials and Methods for details. Trace (i) shows the emission from the singly labeled enzyme, CPR-D or CPR-A, excited at 495 nm and 655 nm, respectively. Trace (ii) shows the emission from CPR-DA exited at 495 nm. Trace (iii) shows the subtraction of trace (i) from trace (ii) to give the change due in emission due to FRET only. Residuals for each fit (A and B) are shown below the respective panel and have the corresponding color. The residuals are essentially randomly distributed over the time range. Conditions: 0.4 µM CPR-DA, 50 mM potassium phosphate, pH 7 at 25°C and 5 mM NADPH. (TIF) [file pbio.1001222.s007.tif]

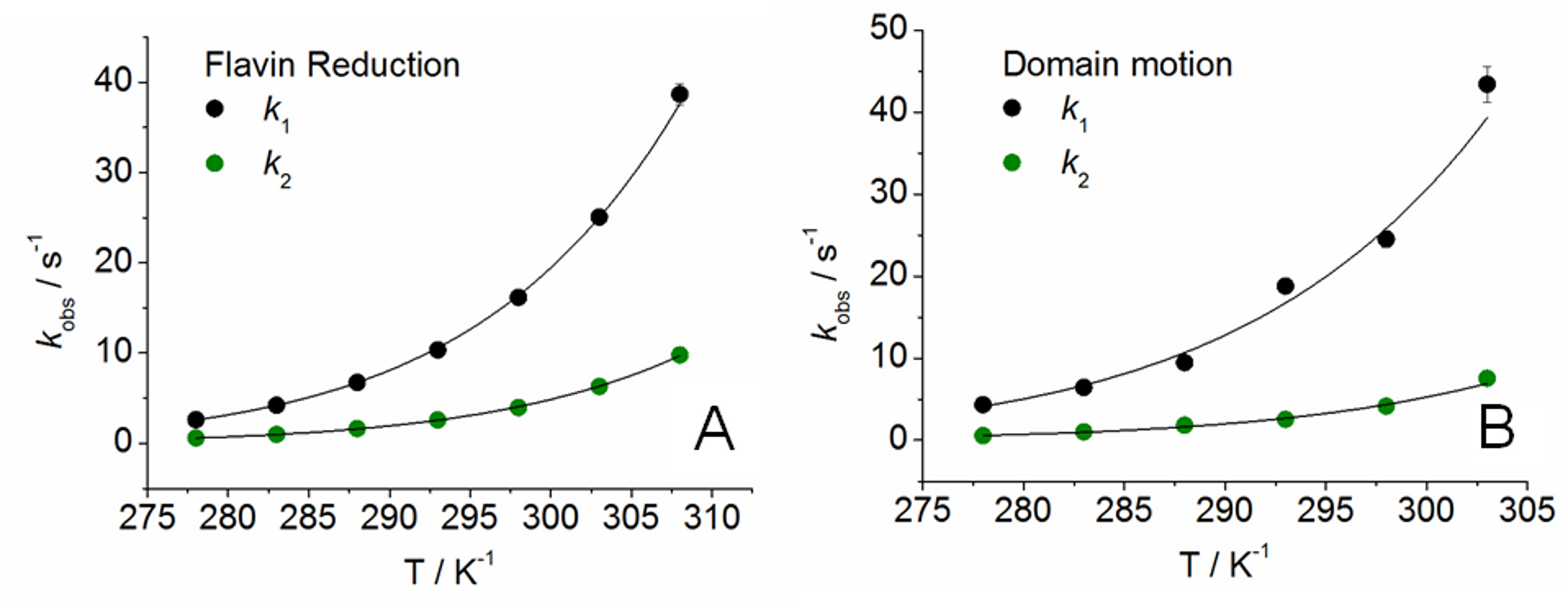

Supplement: Figure S10 — Temperature-dependence of the observed rate of flavin reduction (A) and domain motion (B) fit to the Marcus equation (Text S1, Equation S2). The values for k obs were extracted as described in Materials and Methods and are only given for the first two kinetic phases. The resulting parameters from fitting to the Marcus equation are given below (Table S2). Conditions: 50 mM potassium phosphate pH 7, 0.5 µM CPR-DA and 5 mM NADPH. (TIF) [file pbio.1001222.s010.tif]

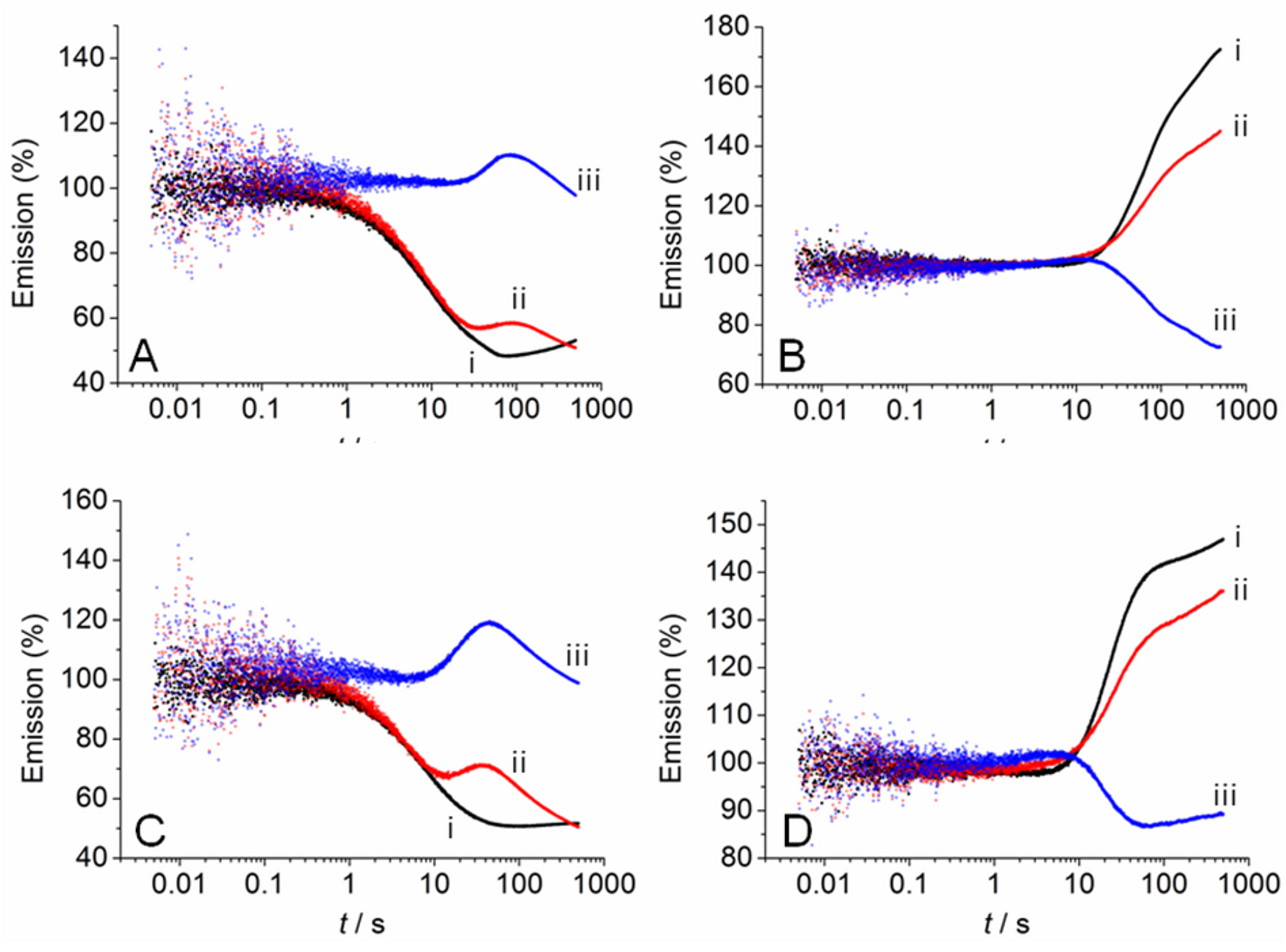

Supplement: Figure S11 — Example stopped-flow transients donor (A/C) and acceptor (B/D) emission upon flavin reduction with sodium dithionite in the absence (A/B) or presence (C/D) of bound NADP+. The emission is given as percentage change, where t = 0 is 100%; see Materials and Methods for details. Transient (i) shows the emission from the singly labeled enzyme (CPR-D or CPR-A). Transient (ii) shows the emission from CPR-DA exited at 495 nm. Transient (iii) shows the subtraction of transient (i) from transient (ii) to give the change due in emission due to FRET only. Conditions: 0.4 µM CPR-DA, 50 mM potassium phosphate, pH 7 at 20°C and 5 mM NADPH. (TIF) [file pbio.1001222.s011.tif]
