# Supplementary material for: Coupled Motions Direct Electrons along Human Microsomal P450 Chains
Source: PLoS Biol. 2011 Dec 20;9(12):e1001222. doi: 10.1371/journal.pbio.1001222 (PMC3243717; doi:10.1371/journal.pbio.1001222)
Supplement: Table S1 — Donor and Acceptor emission extracted from the trace in Figure S7 and Figure 3A. (DOC) [file pbio.1001222.s012.doc]

**Table S1.** Donor and Acceptor emission extracted from the trace in Figure S7 and Figure 3A.

|  | Donor emission (Figure S7A) | | | Acceptor emission (Figure S7B) | | |
| --- | --- | --- | --- | --- | --- | --- |
|  | CPR-D (i) | CPR-DA (ii) | FRET (iii) | CPR-A (i) | CPR-DA (ii) | FRET (iii) |
| A_1_ | 6.3 ± 0.1 | 2.9 ± 0.4 | -3.3 ± 0.1 | 10.9 ± 0.2 | 6.0 ± 7.8 | -8.3 ± 0.3 |
| *k*_1_ | 22.8 ± 0.4 | 23.9 ± 2.8 | 26.0 ± 1.9 | 26.6 ± 1.6 | 18.3 ± 24.4 | 26.9 ± 1.8 |
| A_2_ | -18.8 ± 0.1 | -6.2 ± 0.4 | 13.1 ± 0.2 | -13.6 ± 0.2 | -7.8 ± 6.5 | 8.6 ± 0.3 |
| *k*_2_ | 4.8 ± 0.1 | 6.3 ± 0.3 | 4.3 ± 0.1 | 4.1 ± 0.3 | 9.0 ± 3.4 | 3.6 ± 0.2 |
| A_3_ | -23.2 ± 0.1 | -24.1 ± 0.1 | -5.3 ± 0.1 | 2.1 ± 0.2 | -13.5 ± 0.6 | -14.5 ± 0.5 |
| *k*_3_ | 0.3 ± 0.01 | 0.2 ± 0.01 | 0.1 ± 0.01 | 0.6 ± 0.1 | 0.2 ± 0.01 | 0.2 ± 0.01 |
| A_4_ | 23.1 ± 0.4 | 17.1 ±0.1 | -2.5 ± 0.1 | 14.7 ± 0.1 | 35.6 ± 0.6 | 19.4 ± 0.5 |
| *k*_4_ | 0.03 ± 0.001 | 0.04 ± 0.001 | 0.01 ± 0.001 | 0.03 ± 0.001 | 0.03 ±0.001 | 0.03 ± 0.001 |

Amplitude is expressed in % and *k* has units of s^-1^.
