# Supplementary material for: Coupled Motions Direct Electrons along Human Microsomal P450 Chains
Source: PLoS Biol. 2011 Dec 20;9(12):e1001222. doi: 10.1371/journal.pbio.1001222 (PMC3243717; doi:10.1371/journal.pbio.1001222)
Supplement: Table S2 — Parameters derived from fitting the Marcus equation to the temperature-dependence of the rate constants in Figure S10. (DOC) [file pbio.1001222.s013.doc]

**Table S2.**  Parameters derived from fitting the Marcus equation to the temperature-dependence of the rate constants in Figure S10.

|  | Flavin reduction | | Conformational change | |
| --- | --- | --- | --- | --- |
|  | *k*_1_ | *k*_2_ | *k*_1_ | *k*_2_ |
| *λ* (kJ mol^-1^) | 259 ± 6 | 271 ± 5 | 257 ± 18 | 284 ± 13 |
| *T*_da_ (cm^-1^) | 149 ± 8 | 140 ± 9 | 172 ± 153 | 282 ± 181 |
