# Supplementary material for: Coupled Motions Direct Electrons along Human Microsomal P450 Chains
Source: PLoS Biol. 2011 Dec 20;9(12):e1001222. doi: 10.1371/journal.pbio.1001222 (PMC3243717; doi:10.1371/journal.pbio.1001222)
Supplement: Text S1 — Supplementary text to the Materials and Methods, and Results and Discussion sections. (DOC) [file pbio.1001222.s014.doc]

**Supplementary Information**

Coupled motions direct electrons along human microsomal P450 chains

Christopher R. Pudney^1^, Basile Khara^1^, Linus O. Johannissen^2^ and Nigel S. Scrutton^1^

^1^Manchester Interdisciplinary Biocentre, Faculty of Life Sciences, University of Manchester, 131 Princess Street, Manchester, M1 7DN, United Kingdom.

^2^Manchester Interdisciplinary Biocentre, School of Chemical Engineering and Analytical Sciences, University of Manchester, 131 Princess Street, Manchester M1 7DN, United Kingdom

**Text S1.**

**Supplement to Materials and Methods**

***Stopped-flow studies and data fitting.*** Reaction traces were fit to Eq S1.


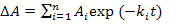
 (S1)

Where *A_i_* is the amplitude and *k_i_* is the rate constant of the *i*th exponential component obtained from the stopped-flow trace and Δ*A* is the total absorbance change. For each fluorescence trace reported in this work the most appropriate fit was found where *n* = 4, *i.e.* four exponential phases unless otherwise stated. For coenzyme studies a saturating concentrations of coenzyme (5 mM) was used. Fluorescence data were fit to a moving average of the emission signal comprising 9 sequential data points. To extract the fluorescence change associated with FRET alone, the percentage of emission from the individual fluorophores (CPR-D/CPR-A) was subtracted from the percentage emission of the corresponding fluorophore in a FRET pair (CPR-DA) where the donor fluorophore was excited. This correction applies to both stopped-flow and static fluorescence measurements.

***Mass spectral analysis.*** Mass spectral analysis was performed at the Protein & Nucleic Acid Chemistry Laboratory, University of Leicester, UK. *Protein Digestion.* The digestion protocol used was a modification of the method described by Chen *et al*  [1]. Briefly 200 *µ*g of freeze-dried protein was dissolved in 50 *µ*l of solution containing 8 M urea/ 50 mM ammonium bicarbonate. To this 5 *µ*l of 100 mM DTT (in 50 mM ammonium bicarbonate) was added and the sample incubated at 60 °C for 30 min to facilitate reduction of disulphide bonds. The sample was allowed to cool to room temperature and the reduced disulphide bonds were alkylated by addition of 15 *µ*l of 100 mM iodoacetamide (in 50 mM ammonium bicarbonate) and incubated at room temperature for 30 min in the dark. The sample was diluted by addition of 305 *µ*l of 50 mM ammonium bicarbonate solution followed by addition of 5 *µ*g of trypsin in 25 *µ*l of 50 mM ammonium bicarbonate and incubated at 37 °C overnight. The digestion reaction was stopped by addition of 2 *µ*l of 100% trifluoroacetic acid.

*HPLC chromatography of tryptic peptides.* Peptide separations were performed on a reverse phase Brownlee Column (Aquapore Octyl, RP-300, C8, 7µm 0.2 x 22 cm / Part No 711-0060) attached to Agilent model 1100 HPLC system consisting of a binary pump, solvent degasser, manual Rheodyne injector with a 20 *µ*l sample loop and a diode array detector. The output line from the detector was attached to a Gilson fraction collector (model 201). 20 *µ*l of the digestion solution was loaded on to the column, pre-equilibrated in 98 % Solvent A (0.1 % TFA/ 2 % Acetonitrile/ 98 % water) and 2 % Solvent B (0.1 % TFA/ 80 % Acetonitrile/ 20 % water) at a flow rate of 0.4 ml/min. The column was developed with a linear gradient of increasing acetonitrile concentration from 2 min to 50 min going from 2 % solvent B to 100 % solvent B and 1min fractions were collected. The diode array detector was set to collect absorbance data at 215 nm, 280 nm, 475 nm and 525 nm.

*Analysis by LC-MS/MS.* LC-MS/MS was carried out using an RSLCnano HPLC system (Dionex, UK) and an LTQ-Orbitrap-Velos mass spectrometer (Thermo Scientific). Samples were loaded at high flow rate onto a reverse-phase trap column (0.3 mm i.d. x 1 mm), containing 5 *μ*m C18 300 Å Acclaim PepMap media (Dionex) maintained at a temperature of 37 °C. The loading buffer was 0.1 % formic acid / 0.05 % trifluoroacetic acid in water.

Peptides were eluted from the trap column at a flow rate of 0.3 *µ*l/min and passed through a reverse-phase capillary column (75 *μ*m i.d. x 250 mm) containing Symmetry C18 100 Å media (Waters, UK) that was manufactured in-house using a high pressure packing device (Proxeon Biosystems, Denmark). The output from the column was sprayed directly into the nanospray ion source of the LTQ-Orbitrap-Velos mass spectrometer.

The LTQ-Orbitrap-Velos mass spectrometer was set to acquire a 2 microscan FTMS scan event at 30000 resolution over the m/z range 400-1800 Da in positive ion mode. Accurate calibration of the FTMS scan was achieved using a background ion lock mass for polydimethylcyclosiloxane (445.120025 Da). Subsequently, up to 20 data dependent CID MS/MS were triggered from the FTMS scan and performed in the LTQ-Velos ion-trap. The isolation width was 2.5 Da, normalized collision energy 35.0, Activation Q 0.25, Activation time 10 ms. Dynamic exclusion was enabled.

*Data Analysis.* The .raw data file obtained from each LC-MS/MS acquisition was processed using the Raw2MSM application [2]. Each file was in turn searched using Mascot [3] (version 2.2.04, Matrix Science Ltd.) against a custom database into which the sequence had been inserted. The peptide tolerance was set to 5 ppm and the MS/MS tolerance was set to 0.6 Da. Variable modifications were set as Alexa488, Alexa488-Na, carbamidomethyl cysteine, oxidation pyro-Glu and pyro-carbamidomethyl cysteine. The enzyme was set to Trypsin/P and up to 3 missed cleavages were allowed. A decoy database search was performed. Peptides found to contain the Alexa488 or Alexa488-Na modification were inspected manually for validation of site localization.

***CPR variant expression and purification.*** The C228S variant CPR enzyme was isolated using the QuikChange mutagenesis protocol (Stratagene) using the following oligonucleotide: 5'- TGG CCG GCC GTG AGT GAA CAC TTT GGG -3'. The mutant gene was completely sequenced to confirm the integrity of the construct. Expression and purification of the mutant CPR enzyme was as described for the wild-type enzyme.

**Supplement to Results and Discussion**

***An experimental system that reports directly on CPR domain motion*.** We have generated homology models of open and closed human CPR based on available X-ray crystal structures of rat CPR. An X-ray crystal structure of human CPR is available [4]. Our homology model for the closed CPR is essentially identical to that of the published human CPR structure. In the present study, we have used our homology models as the human structure is only available in the closed form.

We have labelled CPR with a pair of extrinsic fluorophores which give a measurable FRET signal. The Alexa 488/Cy 5 FRET pair is a good choice due to stability of the dyes, high quantum yield, good spectral overlap and good separation of emission peaks. The Förster distance for this FRET pair is ~ 49 Å, similar to the inter-cysteine distance of C228 – C472 (51.2 Å) and C228 – C566 (57.3 Å) in CPR assessed from our closed CPR homology model. This means the observed fluorescence emission signals are large enough to accurately monitor even subtle changes in donor-acceptor fluorophore fluorescence, but also the emission of the donor and acceptor can be separated accurately using optical filters. From our open homology model the inter-cysteine distance is decreased for both the C228-C472 (46.9 Å) and C228-C566 (51.5 Å), an increase of 4.3 Å an 5.6 Å, respectively.

Figure S1 shows the absorbance spectra of CPR-DA, demonstrating that the donor, Alexa 488, and acceptor, Cy 5, fluorophores are bound at a 1:1 ratio. We note that it is possible to alter this ratio, but the total number of molar equivalents of extrinsic fluorophore remains 2. Mass spectral analysis (Figure S2) suggests three cysteine residues are labelled, C228, C472 and C566. That, two molar equivalents of dye are bound at 3 cysteine residues suggests there is fractional labelling of each of the cysteine residues. Peptide fragments extracted by HPLC chromatography showed significant overlap in their elution profiles making assignment of the peaks in Figure S2 to specific peptide fragments difficult. However, we note that the C228S CPR variant (discussed below) gives ~1.4 molar equivalents of the extrinsic fluorophore bound to CPR, meaning nearly a third of bound fluorophores are positioned at C288 in the FMN domain. This relatively high labelling means variation in the FRET signal arising from changes in the FMN/FAD interdomain distance give rise to a significant change in the observed emission intensity.

Below we provide additional controls that demonstrate FRET in this system reports on conformational change only. Figure S3A shows how the emission of Cy 5 changes when bound to CPR. Figure S3B shows that excitation at 495 nm (donor excitation wavelength) does not give rise to a significant Cy 5 emission peak and is far smaller than the signal observed for Cy 5 emission when in the Alexa 488-Cy 5 FRET pair (~ 3 %). As such the emission observed for Cy 5 due to FRET is not complicated by the intrinsic fluorescence of Cy 5. Further this signal is accounted for in the deconvolution of the FRET response as described in *Materials and Methods*.

Figure S4A shows the emission spectra of an equimolar mixture of CPR-D and acceptor labelled CPR (CPR-A). If there is a stable protein-protein interaction (such as dimer formation), we would expect to observe significant emission from Cy 5 following excitation of Alexa 488 due to FRET from one molecule of CPR to the other. Figure S4A *inset* shows that there is a very small amount of emission around the emission peak of Cy 5. The magnitude of this emission is consistent with the small signal at ~665 nm from exciting CPR-A alone at 495 nm as is shown in Figure S3B. These data then demonstrate that there is no inter-protein FRET. Moreover, these data suggest that there is no stable higher order structure formation such as a dimer.

Based on the position of the extrinsic fluorophores shown in Figure 1 of the main manuscript we do not expect a direct interaction between the flavin moieties and Alexa 488/Cy 5. However, if there is a significant physical interaction between the extrinsic fluorophores and the flavin moieties, we expect this to manifest as a change in the flavin emission. The black line in Figure S4B shows the emission spectra of CPR where the flavin moieties are excited (455 nm), giving rise to a characteristic flavin emission centred at ~550 nm. By monitoring the flavin emission of CPR-A it is possible to examine the flavin emission with an extrinsic fluorophore attached uncomplicated by overlapping fluorescence from the fluorophore. The red line in Figure S4B shows that excitation of CPR-A at 455 nm gives rise to a similar flavin emission peak as CPR alone. That the flavin peaks are so similar argues there is no significant physical interaction between the flavin moieties and the extrinsic fluorophores. Whilst the flavin emission in Figure S4B is very similar it is not identical. Comparing the ratio of the peak integrals for flavin and Cy 5 emission with and without Cy 5 bound (Figure S4B) gives a ratio of 0.87 for the flavin emission (decrease when Cy 5 bound) and 1.14 for Cy 5 emission (increase when Cy 5 bound). This is the expected pattern due to FRET from the flavin moieties to Cy 5 and is the cause of the slight variance between the flavin emission. The magnitude of this change is very small, being < 1 % of the Cy 5 emission attributable to FRET from Alexa 488 to Cy 5 and so does not complicate our FRET data. Again this signal is accounted for in the deconvolution of the FRET response as described in *Materials and Methods*.

These data provide strong evidence that our experimental system can report on conformational change in CPR and that any changes we observe in the emission of the extrinsic fluorophores are not due to direct interaction with the reactive groups or stable protein-protein interactions. As such we are confident that we are able to ascribe changes in fluorescence emission of both Alexa 488 and Cy 5 to conformational changes in CPR.

From Figure S5A it appears that there is significant quenching of the fluorophores as NADP^+^ is titrated against CPR. This may arise from direct quenching by NADP^+^, or quenching by aromatic residues as CPR undergoes a conformational change associated with coenzyme binding. The emission from the donor and acceptor arising from FRET (Figure S5B) show an increase in donor emission with a corresponding decrease in acceptor emission. This is as expected for FRET; however the relative magnitude of the change does not track perfectly, with a larger change in the acceptor compared to the donor. As such we treat these data qualitatively, restricting our analysis to relative opening or closing.

***Conformational change occurs on the timescale of chemical turnover.*** We investigated whether there is an observable change in emission of the extrinsic fluorophores following reduction of the flavin cofactors in CPR by NADPH (Figure S7). Reaction traces [Figure S7 trace (i)] show the variation in emission for both of the individual fluorophores, CPR-D (excited at 495 nm) and CPR-A (excited at 655 nm). These fluorescence traces show the changes in emission in the absence of FRET (since the studied enzyme forms contain only one extrinsic fluorophore). There is a large change in the magnitude of emission for both CPR-D and CPR-A that we infer reports on conformational change in the enzyme. We expect the change in emission of the individual fluorophores to be due predominantly to quenching by aromatic amino acid residues. FRET from enzyme bound Alexa 488 to the flavin moieties has been reported in other systems, potentially contributing to the emission in CPR-D [6]. We do not expect a similar contribution from CPR-A since the spectral overlap between the flavins and Cy 5 is very poor. Consequently, we do not necessarily expect the variation in emission of CPR-D and CPR-A during flavin reduction to give identical fluorescence traces. Further, the fluorophores have significant structural variation and therefore will respond differently to a given physiochemical environment. Further, we have monitored the transient state absorbance signal attributable to flavin reduction (600 nm) for the labelled CPR in the same manner as for unlabelled CPR. We find that the extracted observed rate constants for electron transfer are the same. This demonstrates that the binding of the fluorophores does not affect the rate constants for flavin reduction.

Due to the complexity of the system we have preferred to express our data as relative opening and closing of CPR based on the change in FRET efficiency. However, we note that based on the donor emission alone, the extrinsic fluorophore distance changes by ~2.5 Å being 49 Å and 47.5 Å for oxidised and fully reduced CPR, respectively. Given that a decrease in distance between the extrinsic fluorophores reflects a relative opening of CPR, this is consistent with the magnitude of CPR opening expected based on the inter-cysteine distance in the open and closed homology models (~ 5 Å). However, we stress that given the size of the fluorophores and the complexity of the system the two values are not necessarily expected to be the same.

Our homology models of different open/closed states of CPR suggest that there is very little change in distance between C472/C566 in the FAD domain, but a significant change in distance between C228 and C472/C566. We wished to confirm this and access directly the effect of any FAD domain conformational change on the time-resolved FRET measurements. As such, we have monitored the time-resolved FRET response of the C228S CPR variant as above with the results shown in Figure S8. The FRET response in this enzyme variant cannot report on changes in distance between the FMN and FAD domains but instead provides a control for localised FAD domain motion associated with NADPH binding and subsequent CPR reduction. The extracted time-resolved A:D emission ratio (Figure S8) suggests there is a small conformational change in the FAD domain which correlates with both 2- and 4- electron reduction. The key finding however, is that the deconvolution of these changes in A:D from the observed changes in A:D for wild-type CPR suggest the FAD conformational change has a negligible effect on the observed A:D trend as expected (Figure S9). These data confirm that the time resolved FRET response we monitor in the wild-type enzyme reflects CPR opening and closing.

***CPR exhibits redox-state dependent conformational change.*** Analysis of the temperature dependence of observed rate constants associated with an electron transfer event has been used as a to indicate a true electron transfer reaction (*k*_obs_ = *k*_ET_) or one gated (rate limited) by a non-electron transfer event such as conformational change [6]. The temperature-dependence of “true” electron transfer reactions are best fit using the Marcus equation (*SI* Eq S2), whereas gated electron transfer reactions are best fit using the Eyring equation (Figure 4 main manuscript). The temperature-dependence of the observed rate constants for flavin reduction and conformational change fit to the Marcus equation are shown in Figure S10. Evidence for gated electron transfer can then be inferred from anomalous fitting parameters when fitting temperature-dependence data to the Marcus equation (Table S2). A more detailed description of such deviations is given in ref 5.

The observed electron transfer rate constant depends on the driving force -Δ*G*^o^, the electronic coupling constant *T*_da_ and the reorganization energy *λ*.

(Eq S2)

where *k*_B_ is the Boltzmann constant, *ħ* is Planck`s constant *h* divided by 2*π*. To analyze the temperature dependencies by the Marcus equation (Eq S2, Figure S10, Table S2), the driving force was fixed to zero, since it cannot be obtained reliably from the fit. This assumption is reasonably accurate as demonstrated by Davidson [6]. That is, an uncertainty in Δ*E*^o^ of ± 100 mV results in changes of *T*_da_ < 5 % and in an error of *λ* of ~ 2 x Δ*E*^o^.

It is possible to fit our temperature-dependence data to the Marcus equation for electron transfer as shown in Figure S10. Such an analysis can provide evidence for pure electron transfer or electron transfer which is gated. Gated electron transfer reactions are expected to give anomalous Marcus parameters (large electronic coupling, *T*_da_ > 80 cm^-1^ and very large reorganization energy, (*λ*). We have fit our temperature-dependence data for both *k*_1_ *and k*_2_ for flavin reduction and *k*_1_ *and k*_2_ for the domain dynamics as shown in Figure S10 with the resulting parameters given in Table S2. We find that in each case the value for *T*_da_ is large (well above > 80 cm^-1^) and *λ* is very high (~250 kJ mol^-1^), suggesting that none of our observed rate constants reflect true electron transfer but include some chemical or conformational gating.

**References**

1. Chen EI, et al. (2007) Optimization of Mass Spectrometry Compatible Surfactants for Shotgun Proteomics. *J Proteome Res*. 6:2529–2538.

2. Olsen JV, et al. (2005) Parts per million mass accuracy on an Orbitrap mass spectrometer via lock mass injection into a C-trap. *Molecular & cellular proteomics : MCP* 4:2010-2021.

3. Perkin DN, et al. (1999) Probability-based protein identification by searching sequence databases using mass spectrometry data. *Electrophoresis* 20:3551-3567.4. Xia C, et al. (2011)Structural basis for human NADPHcytochrome P450 oxidoreductase deficiency. *Proc Natl Acad Sci USA* 108:13486-13491

5. Westphal AH, et al. (2006) Real-time enzyme dynamics illustrated with fluorescence spectroscopy of p-hydroxybenzoate hydroxylase. *J Biol Chem* 281:11074-11081.

6. Davidson VL (1996) Unraveling the kinetic complexity of interprotein electron transfer reactions. *Biochemistry* 35:14035-14039.
